# Photothermal responsive porous hollow microneedles as Chinese medicine versatile delivery system for wound healing

**DOI:** 10.1002/SMMD.20240007

**Published:** 2024-07-01

**Authors:** Wanyue Zhang, Lijun Cai, Jingjing Gan, Yuanjin Zhao

**Affiliations:** ^1^ Department of Rheumatology and Immunology Nanjing Drum Tower Hospital School of Biological Science and Medical Engineering Southeast University Nanjing China; ^2^ Oujiang Laboratory (Zhejiang Lab for Regenerative Medicine, Vision and Brain Health) Wenzhou Institute University of Chinese Academy of Sciences Wenzhou Zhejiang China; ^3^ Shenzhen Research Institute Southeast University Shenzhen China

**Keywords:** antibacterial, Chinese medicine, drug delivery, microneedles, wound healing

## Abstract

Chinese medicine is identified as a candidate for wound healing. Attempts in this field tend to develop efficient dosage forms for delivering Chinese medicine with low side effects. In this paper, we proposed novel photothermal responsive porous hollow microneedles (PRPH‐MNs) as a versatile Chinese medicine delivery system for efficient antibacterial wound treatment. The PRPH‐MNs are composed of porous resin shells with good mechanical property, hydrogel cores, and a photothermal graphene oxide hybrid substrate. The hollow structure provides sufficient space for loading the drug dispersed hydrogel, while the porous resin shells could not only block the direct contact between drugs and wound sites but also provide channels for facilitating the drug release from the core. In addition, benefiting from the photothermal effect of their substrate, the PRPH‐MNs could be heated under near‐infrared (NIR) irradiation for controllable promotion of drug release. Based on these features, we have proved that the antibacterial Chinese medicine Rhein loaded PRPH‐MNs were effective in promoting wound healing due to their good antibacterial property and on‐demand drug release. Thus, we believe that the proposed PRPH‐MNs are valuable for delivery of different drugs for clinical applications.

## Graphical abstract

Porous and hollow structure is integrated with photothermal responsive microneedles, providing a large volume for drug loading and more pathways for controllable drug release. The introduction of Chinese medicine avoids side effects and drug resistance with long‐term use. The microneedles loaded with Rhein effectively inhibit bacterial growth and serve as a sustained drug delivery system for effective wound healing.

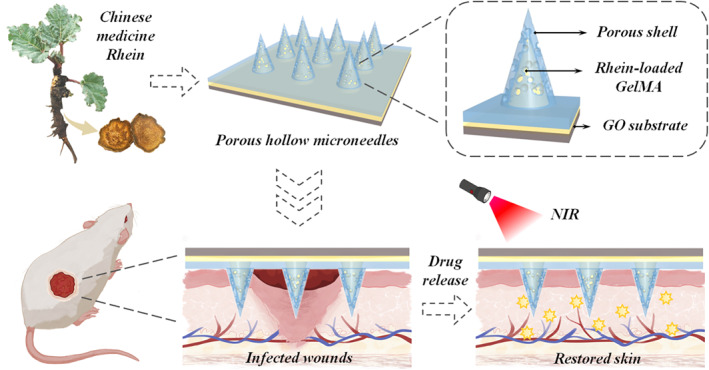

**Key points**

- Porous and hollow structure was integrated with photothermal responsive microneedles, providing large volume for drug loading and more pathways for controllable drug release.
- Introducing Chinese medicine into photothermal responsive porous hollow microneedles effectively avoids side effects and drug resistance with long‐term use.
- Photothermal responsive porous hollow microneedles loaded with Rhein effectively inhibit bacterial growth and serve as sustained drug delivery system for effective wound healing.

## 1 INTRODUCTION

Wound healing has raised worldwide concern due to the complexity and high incidence of wounds.^1^, ^2^, ^3^, ^4^, ^5^ Bacterial infection is regarded as one of the major hurdles that hinders wound healing.^6^, ^7^, ^8^ Up to date, numerous drugs have been applied for wounds aiming at reducing the bacterial infection, which can be mainly divided into western drugs^9^, ^10^ and Chinese medicine.^11^, ^12^ Although western drugs have exhibited good bactericidal properties, most of them have side effects and are prone to cause drug resistance with long‐term use.^13^, ^14^ In contrast, Chinese medicine guarantee effective bacterial inhibition with fewer side effects and flexible dosing, serving as ideal drugs for wound healing.^11^, ^12^, ^15^, ^16^, ^17^ Attracted by this, various dosage forms have been developed to deliver Chinese medicines to the wound sites for antimicrobial purposes to promote wound healing. Although with much progress, the clinical applications of these dosages based on Chinese medicine are still limited because most of the available dosages relied on direct contact of excessive Chinese medicine with wound sites, which may cause inflammatory reactions. Therefore, emerging needs have been raised to develop new dosage forms to deliver Chinese medicine on‐demand for efficient wound healing.

**FIGURE 1:**
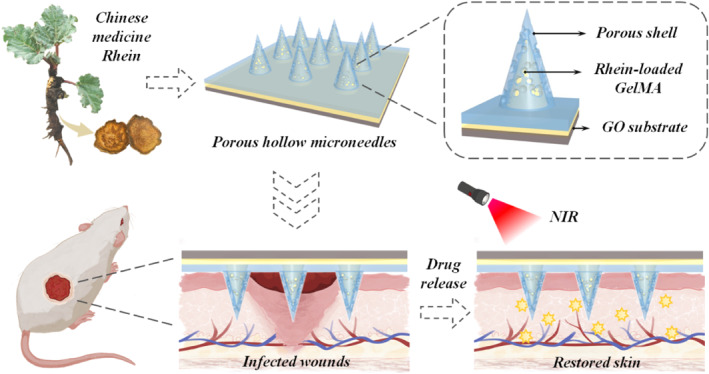
Schematic diagram of Rhein‐loaded PRPH‐MNs and its application for healing wounds.

Herein, we proposed novel photothermal responsive porous hollow microneedles (PRPH‐MNs) as Chinese medicine versatile delivery systems for efficient antibacterial in wounds, as shown in Figure 1. Microneedle is an emerging painless drug delivery dosage form with high patient compliance.^18^, ^19^, ^20^ By integration with functional materials and structure design, these microneedles have been imparted with diverse functions.^21^, ^22^, ^23^ In particular, when microneedles combine with responsive functional materials (*e.g.* graphene oxide, GO), they can realize controllable drug release, showing great potential in wound healing.^24^, ^25^, ^26^, ^27^ In contrast, both hollow and porous structures exhibited outstanding performance in drug delivery because they provide large volumes for drug loading.^28^, ^29^, ^30^ Besides, the porous structure provides more pathways for drug release, serving as a promising architecture for drug delivery.^30^, ^31^, ^32^ Therefore, integrating responsive microneedles with hollow and porous structures is expected to provide possible options for efficient on‐demand drug release.

In this paper, we fabricated the desired PRPH‐MNs through multi‐step replica molding. The PRPH‐MNs are composed of hard porous shells (ethoxylated trimethylopropane triacrylate, ETPTA), hydrogel cores (Rhein‐loaded GelMA (methacrylate gelatin)) for drug loading, and a photothermal substrate (PEGDA (poly(ethylene glycol) diacrylate) mixed with GO). The ETPTA shell not only imparted the PRPH‐MNs with good mechanical property but also blocked the direct contact between drugs and wound sites. The hollow structure provided sufficient space for loading the drug‐loaded hydrogel, endowing the PRPH‐MNs with long‐term drug delivery capability. Notably, the porous structure of the shell, which was formed due to the melting of microfluidic gelatin microspheres, could facilitate the drug release from the core. In addition, due to the photothermal effect of GO, the PRPH‐MNs could be heated under near‐infrared I (NIR‐I) irradiation for promoting the drug release. Based on these features, we loaded the PRPH‐MNs with Rhein, a kind of antibacterial Chinese medicine, to evaluate their performance in diabetic wound healing. Results showed that these Rhein‐loaded PRPH‐MNs could promote the wound healing benefiting from their good antibacterial property, and on‐demand drug release, offering efficient candidates for clinical applications.

## 2 MATERIALS AND METHODS

### 2.1 Materials and animals

Gelatin (GT, from porcine skin), 2‐hydroxy‐2‐methylpropiophenone (HMPP), ETPTA, PEGDA, and GO dispersion were bought from Sigma‐Aldrich. Rhein was bought from Xi'an Yunyue Bio Tech Co., Ltd. 3‐ (4,5‐dimethyl‐2‐thiazolyl)‐2,5‐diphenyl‐2‐H‐tetrazolium bromide (MTT) assay kit was acquired from J&K Scientific Ltd., China. Calcein acetoxymethyl ester (Calcein‐AM) was purchased from Molecular Probes Co., USA. Propidium iodide (PI) and SYTO 9 green fluorescent nucleic acid stain (SYTO) were purchased from Beyotime Biotechnology. Dimethyl sulfoxide (DMSO) was acquired from Aladdin. Phosphate buffer solution (PBS, 1X) was purchased from Servicebio Co. Deionized (DI) water was obtained using a Milli‐Q Plus 185 water purification system (Millipore, Bedford, MA). Fetal bovine serum (FBS), penicillin‐streptomycin double antibiotics, Dulbecco’s modified Eagle medium, trypsin‐ethylenediaminetetraacetic acid (Trypsin‐EDTA) (0.25%, 1X) were obtained from Gibco. Sprague‐Dawley (SD) rats (200–220g) were provided from Jinling Hospital, Nanjing, China. Embryonic fibroblast cells (NIH‐3T3) were obtained by Cell Bank of the Chinese Academy of Sciences, China. Bacterium including *Staphylococcus aureus* (*S. aureus*) and *Escherichia coli* (*E. coli*) were acquired by BeNa Culture Collection. All rats were treated exactly as recommended in the Guide for the Care and Use of Laboratory Animals.

### 2.2 Preparation of electrospray gelatin microspheres

The electrospray device was composed of a high‐voltage power supply, a micro‐injection pump and a capillary‐based nozzle. 5% (w/v) gelatin solution was injected from the capillary‐based nozzle by the micro‐injection pump at flow rates of 5–15 mL h^−1^. A 4.5–9 kV voltage charge was produced by a high‐voltage power supply on the syringe needle to generate gelatin droplets with different sizes. The ejected gelatin microspheres were collected in a small dish containing iced ETPTA to solidify the gelatin droplets, acquiring gelatin microspheres.

### 2.3 Fabrication of PRPH‐MNs

To generate PRPH‐MNs, polydimethylsiloxane (PDMS) was employed to replicate a positive mold adapted to the negative microneedle template. Mixture solution containing ETPTA, HMPP (1%, v/v) and gelatin microspheres was added into the microneedle negative mold, followed by centrifugation to deposit gelatin microspheres at the tip of the microneedle template as well as removing air from the template. Next, a pad of suitable height was settled on the template and a positive mold was utilized to cover the mixture solution on the negative mold. After ultraviolet (UV) exposure, microneedles with hollow structure were obtained by removing the positive mold. Then, DI water at a temperature of 50°C was applied to soak the hollow microneedles to melt the gelatin microspheres. For drug loading, 0.05% (w/v) Rhein and HMPP (1%, v/v) were mixed into pre‐gel solution of 15% (w/v) GelMA. The resultant solution was poured into the cavity of hollow microneedles followed by vacuuming to draw out air and UV polymerization. To endow the drug‐loaded porous hollow microneedles (PH‐MNs) with photothermal responsive property, 30% (v/v) PEGDA 700 containing different concentrations of GO and HMPP (1%, v/v) was covered over the drug‐loaded GelMA layer, followed by UV curing to achieve PRPH‐MNs.

### 2.4 Mechanical performance of PRPH‐MNs

Mechanical performance is an essential indicator for evaluating microneedles. Using a force analyzing instrument to carry out the mechanical characterization. In order to explore the effects of hollow and porous structures on microneedles, ETPTA solid microneedles, porous microneedles, PH‐MNs and PRPH‐MNs were prepared. The microneedles were placed on the platform horizontally. Once the microneedle tips were in contact with the force sensor, the instrument began to record force changes. The experiment was stopped once all the microneedle tips were broken. The sensor speed was set to 5 mm min^−1^.

### 2.5 Photothermal response optimization experiment

The photothermal property of microneedles needs to be optimized for fast and efficient photothermal response and suitable for human skin. 30% (v/v) PEGDA with different concentrations of GO with 30% (v/v) PEGDA were made into square films of 1 × 1 cm^2^. A NIR laser was employed to irradiate the film at a height of 10 cm to make its irradiation cover an area of exactly 1 × 1 cm^2^. The NIR power was controlled by adjusting the current of the laser emitter. The temperature change over time was recorded using an infrared thermal imager. The calculation of average values, error bars and significant difference analysis for all data in this manuscript was conducted using Microsoft Excel. The version used was 16.84 (24,041,420).

### 2.6 Drug release of PRPH‐MNs

Rhodamine B (RhB) was chosen as the small molecule drug model to investigate the pharmacokinetic release. 30 μL 15%, 20%, 25% (w/v) GelMA with 5 mg mL^−1^ RhB were UV polymerized and placed in 1 mL PBS solution, respectively, incubated at 37°C with 450 rpm shaking. Then, 100 μL of the release buffer sample was extracted and collected, followed by adding 100 μL fresh PBS. Samples were taken at 1 h intervals for the first 12 h, and thereafter the sampling interval was increased to be taken at 24, 36, and 48 h. The release of RhB could be analyzed by measuring their fluorescence intensity using a microplate reader. To characterize the drug release promotion effect of photothermal stimulation, RhB‐loaded 15% (w/v) GelMA was tested at 37°C, 42°C and 47°C (emulating no NIR, NIR current 1.4 A and NIR current 1.5 A, respectively).

### 2.7 Evaluation of biocompatibility performance

ETPTA PH‐MNs were washed with 75% alcohol and PBS for three times, respectively, and treated with UV light overnight. Next, PH‐MNs were soaked in culture medium (1% penicillin‐streptomycin double antibiotics, 15% FBS) to obtain the extracts. NIH‐3T3 cells were grown in blank well plates (control group) and cultured with the extracts (experimental group), respectively. The cultivation time was 3 days. At the same time period on each day of the experiment, MTT assay was performed to quantitatively evaluate the cytosafety of ETPTA PH‐MNs. MTT solution (10% v/v) was added in each group and mixed uniformly, followed by the cells incubated with MTT for 4 h. Then, the solution was removed, followed by the addition of 500 μL DMSO to dissolve the formazan. 100 μL DMSO solution was sampled and detected by a microplate reader (SYNEGRY, HTX) to obtain optical density (OD) value. Live cell staining was conducted simultaneously on each day of the experiment. After staining cells with Calcein‐AM (1 μg mL^−1^), a fluorescence microscope was used to record cell morphology and proliferation under 495 nm excitation light.

### 2.8 Antibacterial activity of rhein‐loaded GelMA

To investigate the antibacterial activity of Rhein‐loaded PRPH‐MNs, *S. aureus* and *E. coli* were chosen to construct bacterial models. Four groups were set up in this antibacterial experiment, which were blank control group, PRPH‐MNs group, Rhein‐loaded PRPH‐MNs group, and Rhein‐loaded PRPH‐MNs with NIR treatment group. Bacterial suspensions with a turbidity of 0.5–0.6 (1 × 10^8^ colony‐forming units mL^−1^) were prepared and 500 μL of the suspension was added into each group and incubated at 37°C for 24 h. Group PRPH‐MNs + Rhein + NIR was irradiated with 1.4 A NIR for 10 min in each hour. On the next day, SYTO and Propidium iodide were used to perform bacterial live/dead staining and fluorescent images were recorded for each group.

### 2.9 In Vivo diabetes wound healing experiment

Type I diabetes was induced by intraperitoneal injection of streptozotocin (STZ, 1% w/v, pH = 4.5, dissolved in citrate buffer) in SD rats (70 mg kg^−1^). 3 days later, 10% (w/v) chloral hydrate was injected to anesthetize the rats. Subsequently, two circular wounds with a diameter of 1 cm were created on the back of SD rats. Control group, PRPH‐MNs group, Rhein‐loaded PRPH‐MNs (PRPH‐MNs + Rhein) group without NIR irradiation, Rhein‐loaded PRPH‐MNs with NIR irradiation (PRPH‐MNs + Rhein + NIR) group and direct administration (Rhein) group were set up by randomly dividing the rats into five groups with different interventions on the wounds. The microneedles given were replaced with new ones daily, and the PRPH‐MNs + Rhein + NIR group was treated with 1.4 A NIR irradiation for 30 min every day. Photographs were taken on days 0, 3, 5, 7, and 9 to follow up wound healing. All the rats were sacrificed on day 9, and the granulation tissues in the wound sites were extracted and immersed in paraformaldehyde solution. Hematoxylin‐eosin (H&E) staining was conducted to quantify the epithelial thickness. To assess the collagen deposition and inflammation, Masson staining and immunohistochemical staining for tumor necrosis factor (TNF‐*α*) were performed. For evaluation of neovascularization, tissue vessel density was analyzed by double immunofluorescence staining with platelet endothelial cell adhesion molecule‐1 (PECAM‐1/CD31) and *α*‐smooth muscle actin (*α*‐SMA).

## 3 RESULTS AND DISCUSSION

**FIGURE 2:**
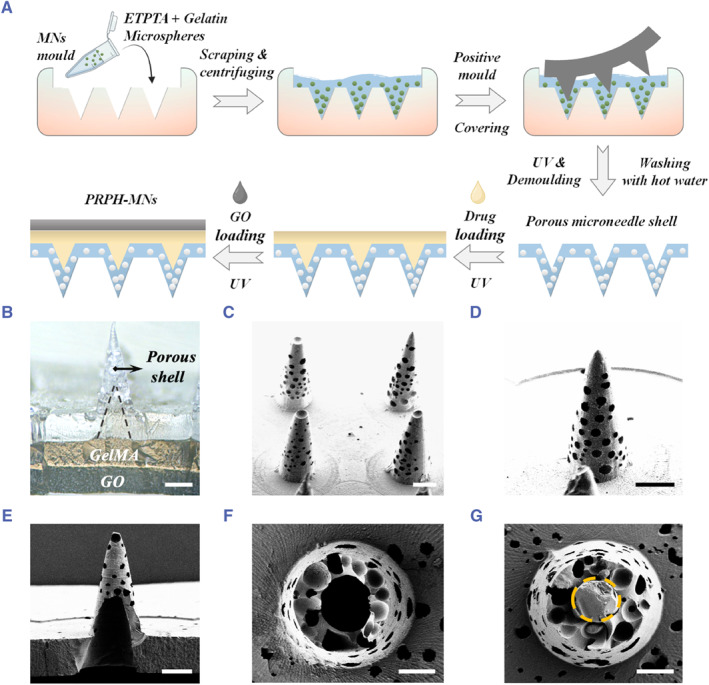
Fabrication process and characterization of PRPH‐MNs. (A) Schematic diagram showing the fabrication process of PRPH‐MNs. (B) Optical image of PRPH‐MNs cross‐section. (C–D) SEM images of PRPH‐MNs (c) and a representative microneedle (D). (E–G) SEM images of magnified cross‐section of porous shell (E, F) and dug‐loaded PRPH‐MNs (G). Scale bars are 200 μm in (B–E) and 100 μm in (F–g).

In a typical experiment, PRPH‐MNs were fabricated through multistep template‐replicating, as shown in Figure 2A. First, a mixture solution containing ETPTA and gelatin microspheres was full‐filled into a negative microneedle mold. Notably, the gelatin microspheres were generated from electrospray technology, whose voltage and flow rate could be adjusted to obtain gelatin microspheres with different sizes (Figure S1). Next, a positive mold was employed to cover the mixture solution in the negative mold. After UV exposure, microneedles with a hollow structure were obtained by removing the positive mold, which provided a large hollow cavity for drug loading. By soaking the hollow microneedles in hot water, hollow microneedles with porous structure were obtained due to the melting of the gelatin microspheres. For drug loading, drugs were mixed into pre‐gel solution of GelMA. The mixture of GelMA pre‐gel solution and drugs was poured into the cavity of hollow microneedles, followed by UV exposure to polymerize the GelMA. To endow the drug‐loaded porous hollow microneedles with photothermal responsive property, a layer of PEGDA containing photothermal responsive GO was applied to cover the drug‐loaded GelMA generating PRPH‐MNs. The obtained PRPH‐MNs are shown in Figure 2B and Figure S2.

To further confirm the architecture of PRPH‐MNs, scanning electron microscopy (SEM) was utilized. According to Figure 2C,D, the obtained PRPH‐MNs exhibited a porous structure. Besides, it was found that a porous shell and large cavity were obtained after the removement of positive mold and melt of gelatin microspheres (Figure 2E,F). Notably, the hollow cavity provided large space for drug loading and the pores were interconnected across the shell, serving as channels for drug release. In addition, after the loading of drug‐loaded GelMA, the cavity was filled (Figure 2G). It is worth mentioning that the porous shell could block the direct contact between the drugs with the external substrate. Overall, the resultant PRPH‐MNs possessed three layers involving a porous hollow shell, drug‐loaded GelMA and a photothermal GO‐contained layer. Furthermore, mechanical tests were carried out. It was demonstrated that these PRPH‐MNs could bear compressing force reaching about 0.4 N per microneedle, which was far exceeding the pressure required for a needle to penetrate the skin, exhibiting great potential for transdermal drug delivery (Figure S3).^33^

**FIGURE 3:**
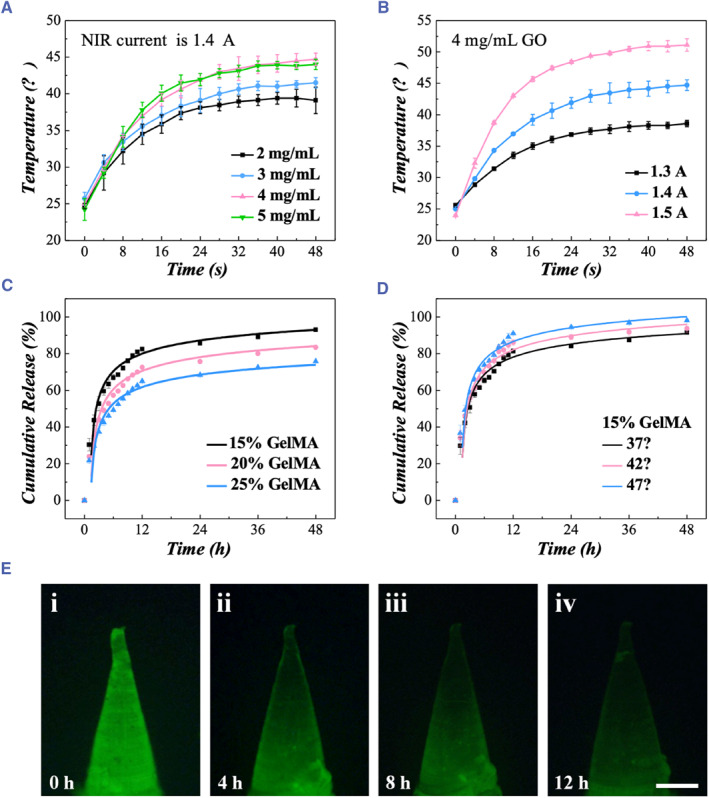
Photothermal response and drug release of PRPH‐MNs. (A) Photothermal performance of PRPH‐MNs with different GO concentrations when NIR current was 1.4 A. (B) Photothermal performance of 4 mg mL^−1^ GO at different NIR currents. (C) Small molecule drug release kinetic curves of GelMA with concentrations of 15%, 20%, 25%. (D) Drug release kinetic curves of 15% GelMA (w/v) with temperature rise. (E) Fluorescence images of RhB‐loaded GelMA microneedle tip at 0 (i), 4 (ii), 8 (iii), 12 (iv) hours of drug release. Scale bar in (E) is 200 μm. NIR, near‐infrared.

Owing to the integration of the GO substrate, PRPH‐MNs were endowed with photothermal responsive property. The photothermal performance of the PRPH‐MNs made from diverse GO concentration under NIR‐I light was evaluated. As shown in Figure 3A, the temperature of PRPH‐MNs increased under the NIR‐I irradiation. Generally, higher GO concentrations contributed to larger temperature changes, while GO concentrations of 4 mg mL^−1^ and 5 mg mL^−1^ had similar temperature response curves. Thus, comprehensively considering both photothermal performance and economical, optimized GO concentration of 4 mg mL^−1^ was selected for the subsequent experiments in this paper. Besides, it is found that higher power of NIR‐I light led to higher temperature changes as well (Figure 3B and Figure S4). Considering that human basal body temperature is around 36.7°C and that high temperature might cause discomfort or even scald, a temperature range of 37–47°C was suitable in this experiment. For PRPH‐MNs with 4 mg mL^−1^ GO, a precise temperature control between 37 and 47°C could be achieved by regulating the irradiation time and the current of the NIR between 1.3 and 1.5 A. Overall, the PRPH‐MNs exhibited desired photothermal responsive property, serving as an ideal candidate for controllable drug release.

Drugs were loaded onto the PRPH‐MNs by mixing them with GelMA to fill the hollow cavity and they could be released from GelMA upon contacting with the tissue fluids. To validate their ability of drug release, these GelMAs were loaded with RhB as a small molecule drug model. As shown in Figure 3E, the initial RhB‐loaded GelMA exhibited a strong fluorescence signal. The fluorescent signal gradually weakened with time, indicating that RhB was gradually released from GelMA. The amount of released RhB was tracked by measuring the fluorescence of the surrounding solution because the solution fluorescence intensity was proportional to the concentration of RhB within a certain range (Figure S5). Considering different concentrations of GelMA might have an influence on the drug release performance, we recorded the cumulative release rates of GelMA with concentrations of 15%, 20%, and 25% (w/v) in PBS. For all groups, the RhB burst in the first 3 h and then were gradually released. After 48 h, RhB‐loaded GelMA with concentrations of 15%, 20% and 25% reached cumulative release rates of 93%, 84%, and 74%, respectively. This result showed that lower concentration of GelMA contributed to higher cumulative release rate, indicating better drug release property. In view of the fact that GelMA with concentration below 15% was difficult to polymerized, we finally chose 15% (w/v) GelMA for the subsequent experiments. Considering that the photothermal‐derived temperature change could promote the drug release, we then compared the drug release performance of 15% GelMA (w/v) at 37°C, 42°C, 47°C, respectively. As shown in Figure 3D, higher temperature led to higher cumulative release rate, indicating better drug release performance.

**FIGURE 4:**
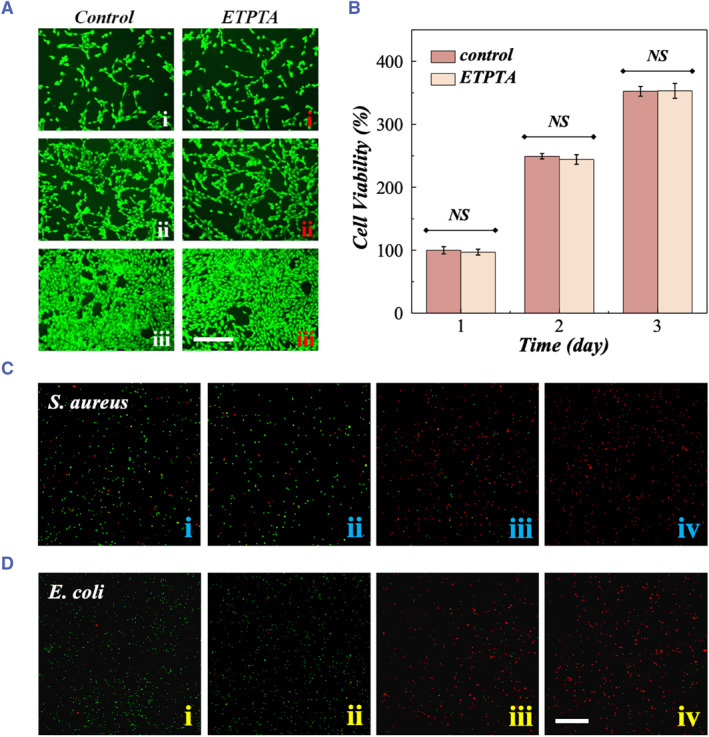
In vitro cytocompatibility experiments on ETPTA and antibacterial experiments. (A) Fluorescence images of NIH‐3T3 cells cultured on day 1 (i), day 2 (ii), and day 3 (iii). (B) Cell viability of Control group and ETPTA group on day 1, 2, 3. (C–D) Live/dead stained images of *S. aureus* (C) and *E. coli* (D) in four groups: (i) Control, (ii) PRPH‐MNs, (iii) PRPH‐MNs + Rhein, (iv) PRPH‐MNs + Rhein + NIR. Significant difference analysis was conducted by two‐tailed Student's *t*‐test. No significance (NS) indicates *p* > 0.05. Scale bars are 100 μm. ETPTA, ethoxylated trimethylopropane triacrylate; NIR, near‐infrared.

Porcine‐derived gelatin, GelMA, PEGDA, and GO, which were used for PRPH‐MN fabrication, are both highly biocompatible materials.^27^, ^34^, ^35^, ^36^ However, the biosafety of ETPTA has rarely been reported. To demonstrate the biocompatibility of PRPH‐MNs, we performed cytocompatibility experiments on ETPTA. As depicted in Figure 4A,B, the relative activity of NIH‐3T3 cells co‐cultured with ETPTA infusion showed the same increasing trend compared to the Control group, which meant that ETPTA material had little influence on cell growth, indicating good biocompatibility. In this experiment, the Chinese medicine Rhein was chosen as the model drug. Extracted from Rheum officinale, Chinese medicine Rhein has a broad‐spectrum inhibitory effect on various bacteria, fungi, and viruses.^37^ Rhein mainly inhibits bacterial growth by restraining bacterial sugar metabolism.^38^ Compared with western drugs, Rhein has fewer side effects and is flexible in the administration, thus supposed to be an ideal drug for wound healing. It was also seen that GelMA could keep Rhein wrapped well and form a microneedle array (Figure S6). To examine the antibacterial performance of Rhein‐loaded PRPH‐MNs, *S. aureus* and *E. coli* were chosen to be specific samples for gram‐negative and gram‐positive bacteria, respectively. As live/dead stained images of bacteria shown in the Figure 4C,D, both Rhein‐loaded PRPH‐MNs without NIR irradiation (PRPH‐MNs + Rhein) and Rhein‐loaded PRPH‐MNs with NIR irradiation (PRPH‐MNs + Rhein + NIR) groups showed fewer living bacteria than the other groups, exhibiting excellent antibacterial effects against both *S. aureus* and *E. coli*, which refer to gram‐positive cocci and gram‐negative bacilli. Fewer viable bacteria were observed after NIR treatment because the temperature rise promoted the release of Rhein from GelMA (Figure S7). In summary, the presented PRPH‐MNs not only showed ideal biocompatibility but also exhibited good antibacterial effect after being loaded with Rhein, which were desirable for applying to wound healing.

**FIGURE 5:**
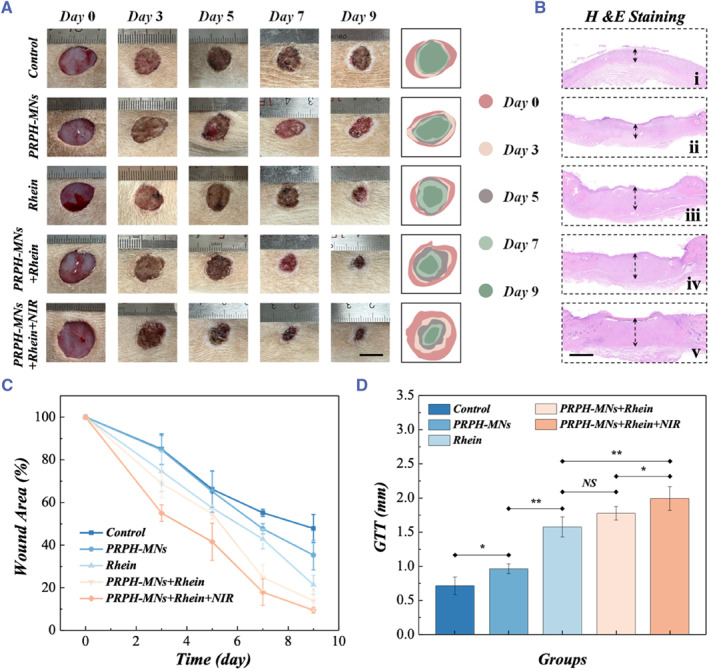
Evaluation of PRPH‐MNs in promoting wound healing in vivo. (A) Representative images recorded during the healing process of skin wounds in five groups, that is, Control group, PRPH‐MNs group, Rhein group, PRPH‐MNs + Rhein group, PRPH‐MNs + Rhein + NIR group. (B) H&E staining of granulation tissues on the 9th day (i, ii, iii, iv, and v represent Control, PRPH‐MNs, Rhein, PRPH‐MNs + Rhein, and PRPH‐MNs + Rhein + NIR, respectively). (C) Quantitative analysis of the wound closure rate within 9 days (D) Data analysis of granulation tissue thickness (GTT) of different groups on day 9. Significances are presented by NS *p* > 0.05, *p < 0.05, and **p < 0.01. Scale bars are 1 cm in (A) and 1 mm in (B). NIR, near‐infrared; NS, No significance.

In view of their special architecture, controllable drug release, good biocompatibility, and antibacterial ability, the Rhein‐loaded PRPH‐MNs were expected to promote wound healing. To validate this, mice with type I diabetic wounds were chosen as diseased models. As shown in Figure S8a, all mice were induced to diabetic mice by intraperitoneal injection of streptozotocin, and then circular wounds of 1 cm in diameter were created on both sides of their backs. Blood glucose of mice before and after diabetes modeling were recorded in Figure S8c, indicating that all mice had abnormal glucose metabolism. It is worth mentioning that the wounds of diabetes modeling were in a chronic inflammation state with decreased self‐repair function. These mice were then divided into five groups for no treatment (Control), blank microneedles (PRPH‐MNs), direct Rhein administration (Rhein), Rhein‐loaded microneedles (PRPH‐MNs + Rhein), and photothermal Rhein‐loaded microneedles (PRPH‐MNs + Rhein + NIR) (Figure S8b). The pictures of wounds were taken on day 0, 3, 5, 7, 9. As shown in Figure 5A,C, the wound areas decreased significantly faster in the PRPH‐MNs + Rhein and PRPH‐MNs + Rhein + NIR groups compared to the Control group and the group given blank microneedles, implying that Rhein‐loaded PRPH‐MNs could promote wound healing. Notably, both PRPH‐MNs + Rhein and PRPH‐MNs + Rhein + NIR groups had a comparable advantage over the direct Rhein administration group since PRPH‐MNs + Rhein not only prolonged the duration of administration but also avoided direct contact of excess Rhein with the wound sites. In addition, the PRPH‐MNs + Rhein + NIR group outperformed the PRPH‐MNs + Rhein group because the temperature rise promoted the Rhein release, consistent with the results of experiments in vitro.

**FIGURE 6:**
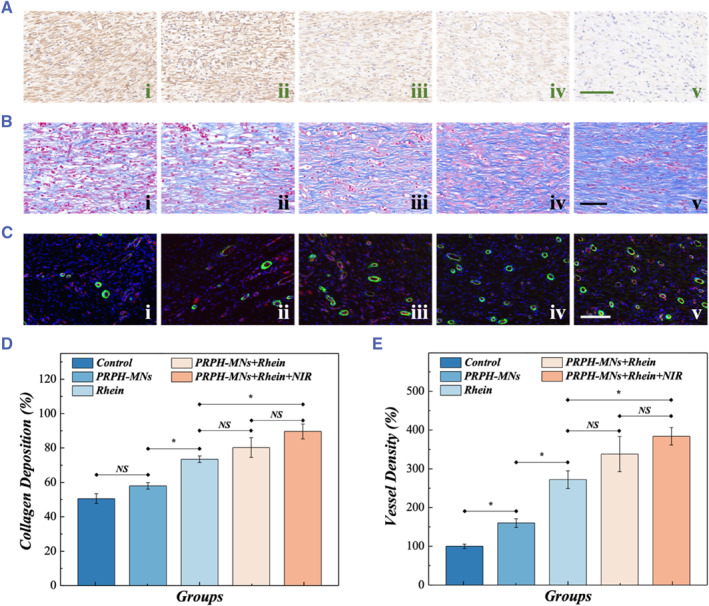
Immunohistochemical, Masson and immunofluorescent staining images of wound tissues in five groups. (A–C), TNF‐*α* immunohistochemistry staining (A), Masson trichrome staining (B), double immunofluorescent staining of CD31 (Red) and *α*‐SMA (Green) (C) images of epithelial tissues in five groups on day 9. (D) Corresponding statistical analysis of collagen deposition fraction in different groups on day 9. (E) Quantification evaluation of blood vessel density in five groups on the 9th day (i, ii, iii, iv, and v represent Control, PRPH‐MNs, Rhein, PRPH‐MNs + Rhein, and PRPH‐MNs + Rhein + NIR, respectively). Significances are presented by NS *p* > 0.05 and *p < 0.05. Scale bars are 100 μm. NIR, near‐infrared; NS, No significance.

For further evaluation of the granulation tissue state, H&E staining was conducted. We found that the wounds of the control group had the thinnest regenerative tissue, while both the granulation tissues and scads of the PRPH‐MNs + Rhein + NIR group were the thickest, indicating that the PRPH‐MNs + Rhein + NIR group exhibited the best curative effect on wound healing (Figure 5B,D). In addition, other characterization methods were also performed, involving immunohistochemical staining of TNF‐α, Masson staining, and immunofluorescence staining (Figure 6A–C). Bacterial infections tend to cause a chronic inflammatory response in diabetic mice. Therefore, effective antibacterial drugs should also improve the inflammatory condition of wounds. TNF‐α, as an endogenous pyrogen, is one of the most important indicators to characterize the inflammatory condition. Generally, high expression of TNF‐α in granulation tissues implies worse wound inflammation. The PRPH‐MNs + Rhein + NIR group showed significantly less yellow inflammatory factors due to the effect of Rhein, which indicated less amount of TNF‐α, confirming that PRPH‐MNs + Rhein + NIR represented good antibacterial effects (Figure 6A). Collagen deposition and alignment can create an active restorative environment for tissue regeneration. According to Figure 6B, there were no significant differences between the PRPH‐MNs group and Control group due to a lack of drug intervention. On the contrary, the collagen of the PRPH‐MNs + Rhein + NIR group showed highly oriented alignment in Masson staining, indicating that it could improve the wound microenvironment and healing conditions. Corresponding collagen deposition fraction statistics were in accordance with the results (Figure 6D). Besides, the PRPH‐MNs + Rhein + NIR group had a greater vascular density and showed better tissue regeneration potential, as shown in Figure 6C,E. All these results suggested that these PRPH‐MNs loaded with Rhein possessed practical value in effective wound repair.

## 4 CONCLUSION

In this paper, we designed a kind of photothermal responsive porous hollow microneedle loaded with Chinese medicine Rhein for controllable drug delivery. This porous hollow microneedle structure was fabricated by multi‐step replica molding, which has good biocompatibility and mechanical properties. This special structure of microneedles solved the pain point issues when delivering Chinese medicine and also integrated the advantages of controllable drug delivery by photothermal materials. It was demonstrated that photothermal responsive porous hollow microneedles loaded with Rhein could effectively inhibit bacterial growth and promote wound healing in *in vivo* experiments. All these results suggest that this novel multifunctional microneedle has promising applications in the field of antibacterial and wound repairing. In addition, the microneedles can be loaded with various medicines, which are expected to be further introduced into the fields of cosmetology and dermatology treatment. Future work is expected to improve this in terms of soft backing material and skin adhesion. The backing of the microneedle patch consists of three layers, among which the ETPTA layer is hard and does not adhere well to the skin. It is conceived that this microneedle will provide new drug delivery strategies for a wide range of application scenarios.

## AUTHOR CONTRIBUTIONS

Yuanjin Zhao conceived the idea and designed the experiment. Wanyue Zhang conducted experiments and data analysis, and wrote the manuscript. Lijun Cai and Jingjing Gan helped with manuscript writing. All authors have read and approved the final manuscript.

## CONFLICT OF INTEREST STATEMENT

The authors declare that there are no competing interests.

## ETHICS STATEMENT

The animal experiments have received approval from the Animal Investigation Ethics Committee of Drum Tower Hospital.

## Supporting information

Supporting Information S1
