# Supplementary material for: Photothermal responsive porous hollow microneedles as Chinese medicine versatile delivery system for wound healing
Source: Smart Med. 2024 Jul 1;3(3):e20240007. doi: 10.1002/SMMD.20240007 (PMC11425051; doi:10.1002/SMMD.20240007)
Supplement: Supplementary file 1 — Supporting Information S1 [file SMMD-3-e20240007-s001.docx]

Supporting Information


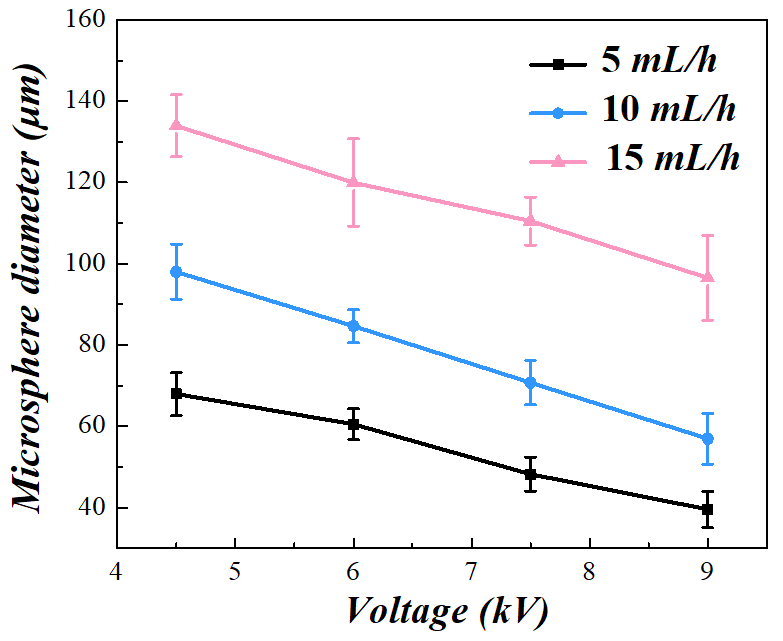


**Figure S1.** Analysis of microsphere diameter at different injection pump flow rate and electrospray DC voltage.


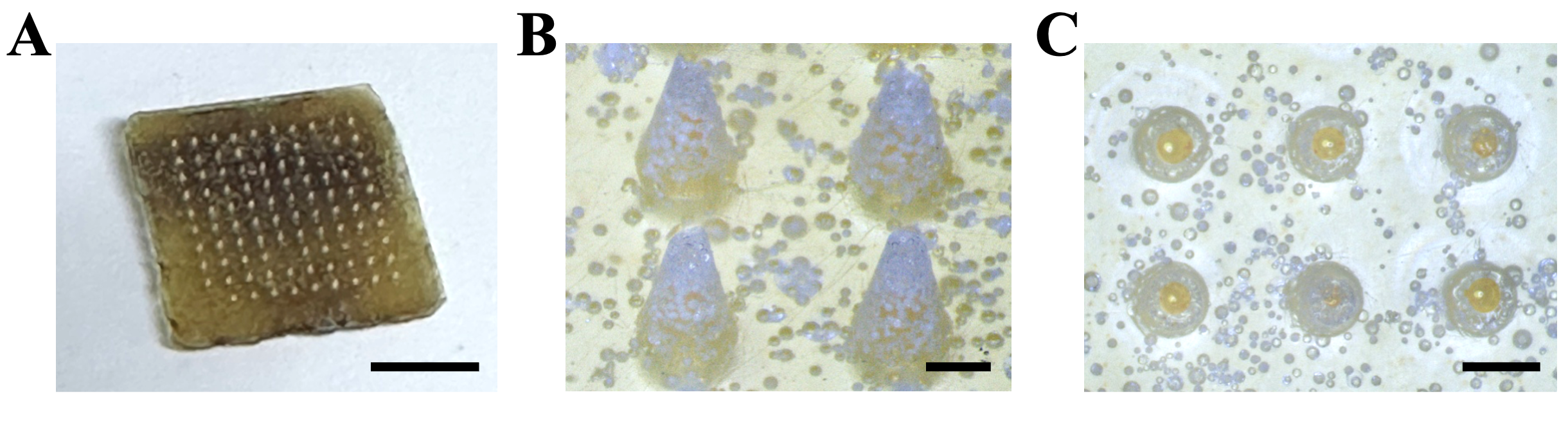


**Figure S2.** (a) Optical image of a PRPH-MN array. (b) Optical image of magnified (c) and cross-section (d) of Rhein-loaded PH-MNs. Scale bars are 0.5 cm in (a), 250 μm in (b) and 500 μm in (c).


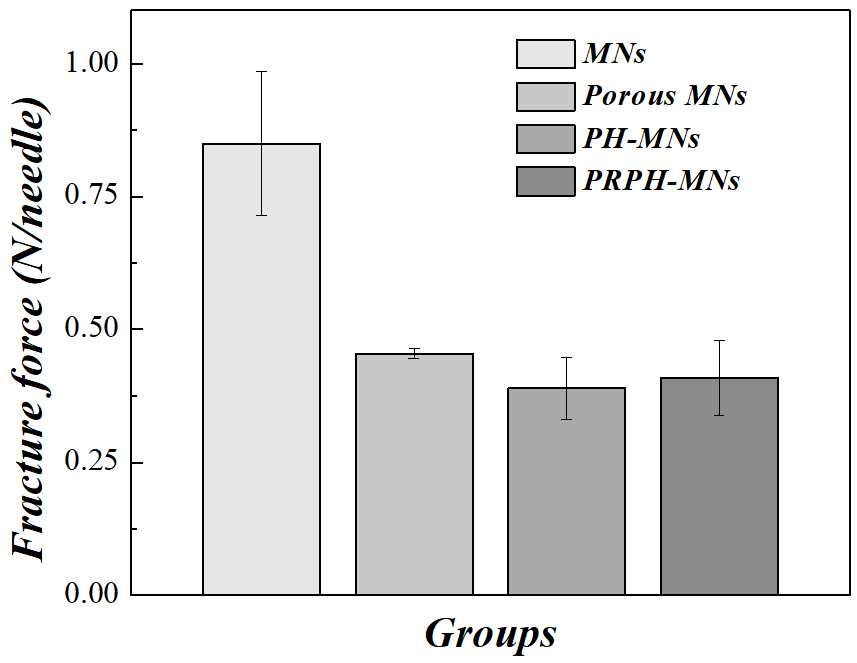


**Figure S3.** Mechanical performance test of ETPTA microneedles (MNs), porous microneedles (Porous MNs), PH-MNs, and PRPH-MNs.


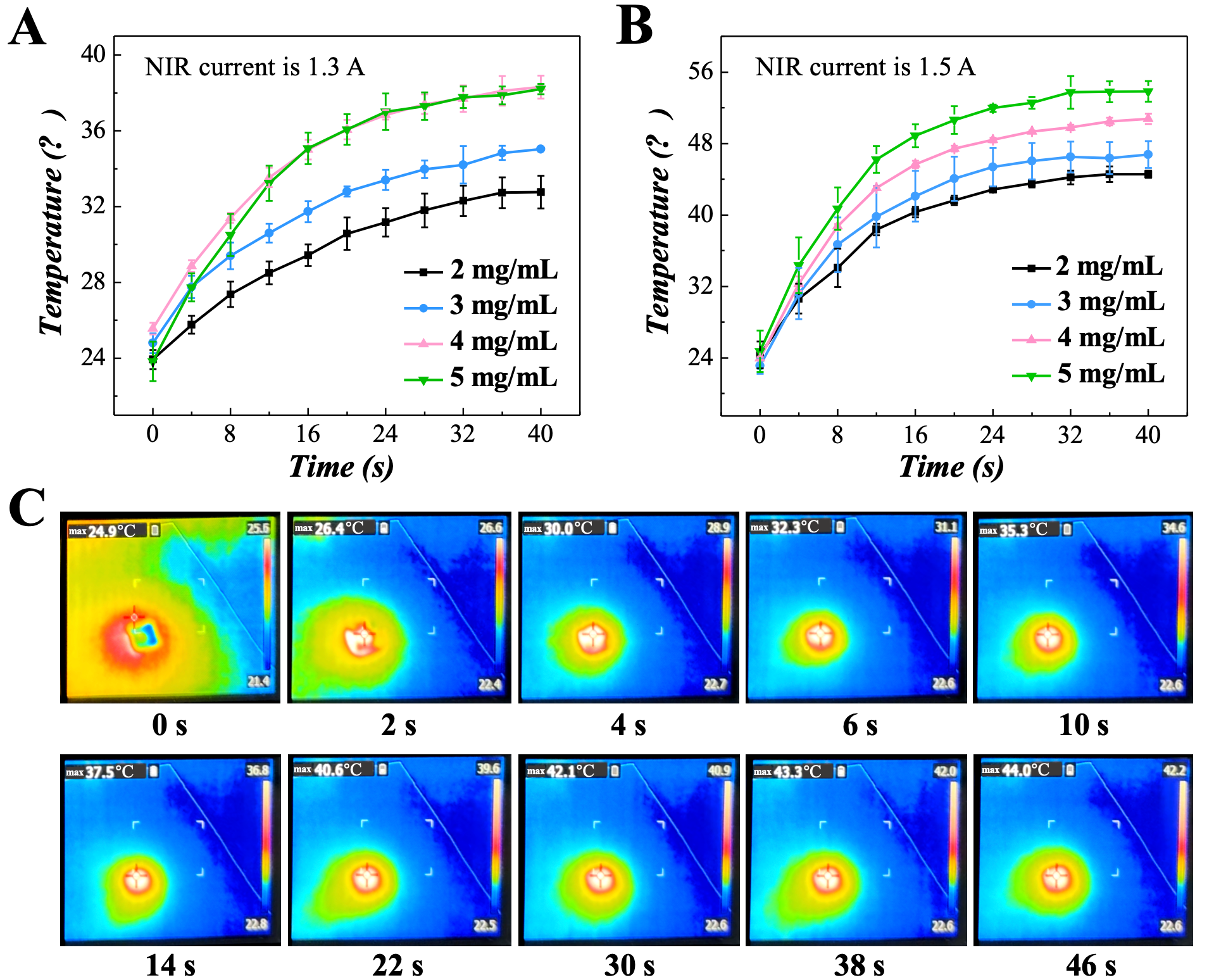


**Figure S4.** (a-b) Photothermal performance of GO with different concentrations when NIR power is 1.3 A (a) and 1.5 A (b). (c) Infrared thermal images of 4 mg mL^−1^ GO warming process at 1.4 A.


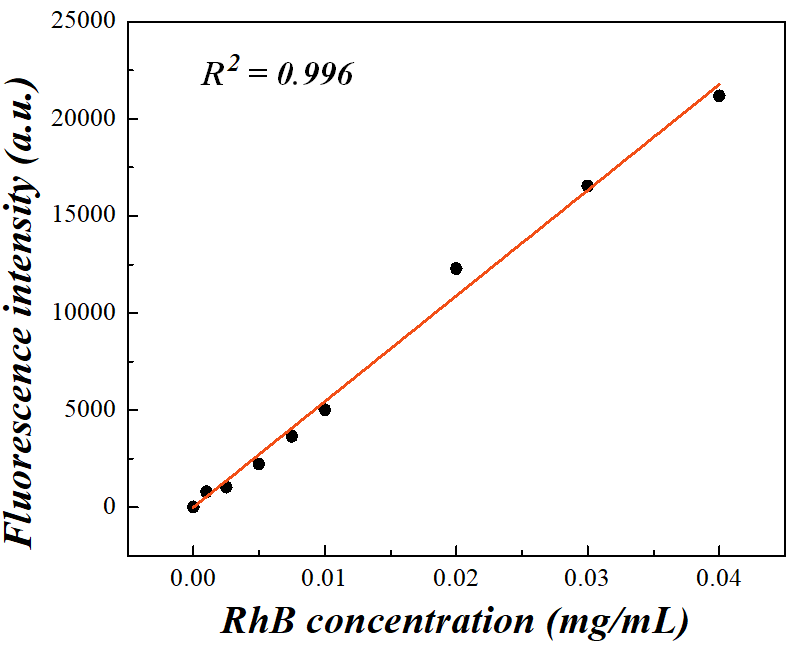


**Figure S5.** Standard curve analysis of RhB concentration and fluorescence intensity.


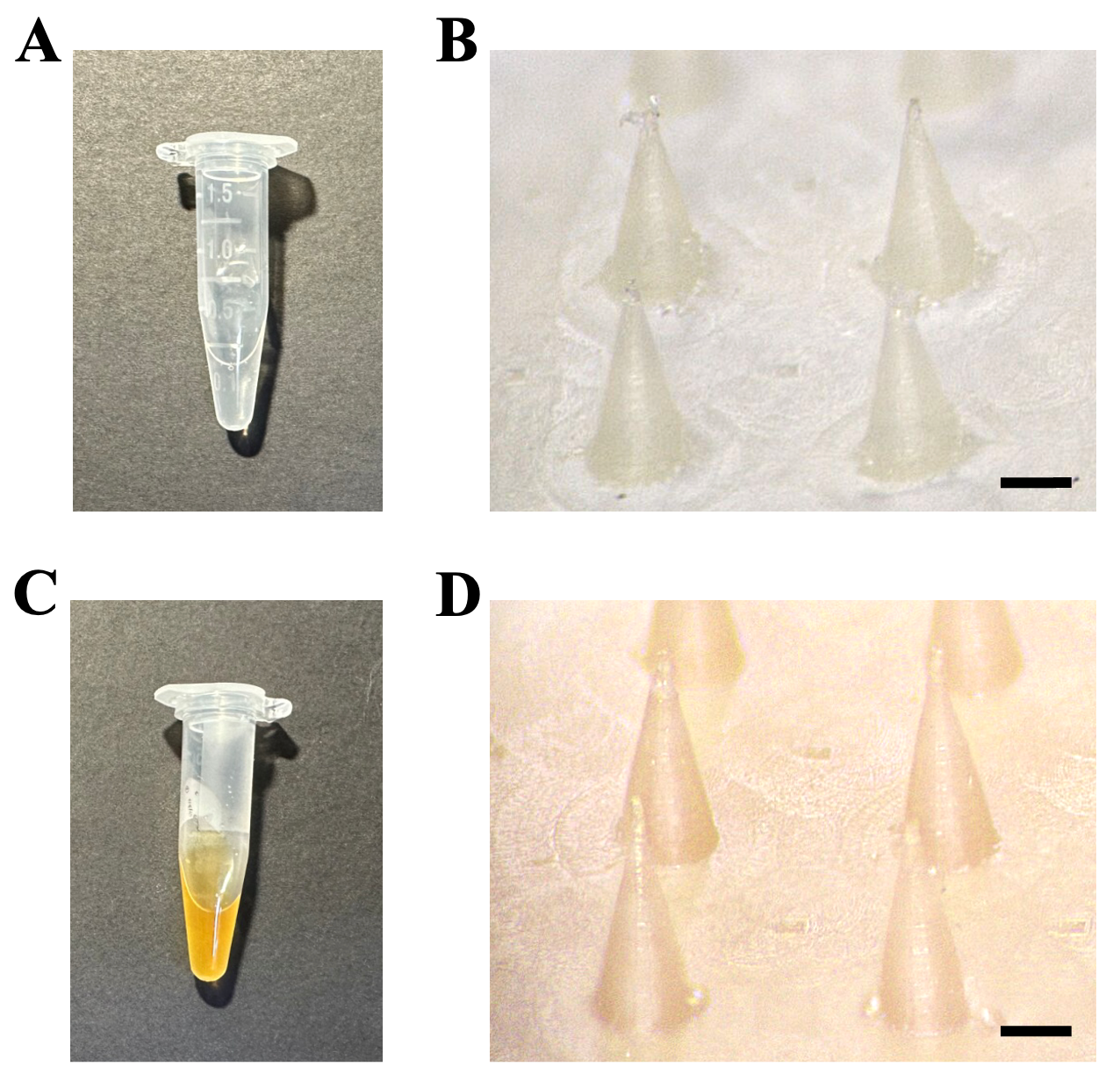


**Figure S6.** (a) 15% (w/v) GelMA solution. (b) GelMA polymerized microneedles. (c) 15% (w/v) GelMA loaded with 0.05% (w/v) Rhein solution. (d) Rhein-loaded GelMA polymerized microneedles. Scale bars are 250 μm.


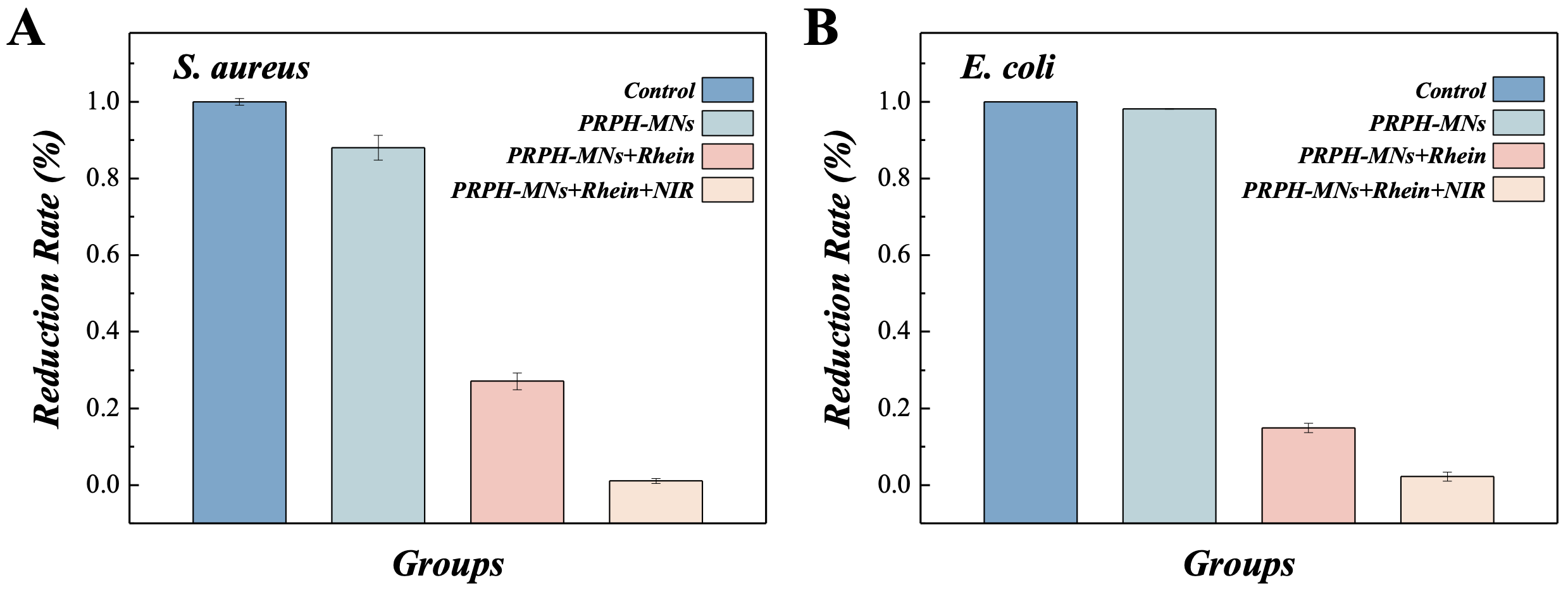


**Figure S7.** (a-b) Quantitative analysis of *S. aureus* (a) and *E. coli* (b) reduction rates in four groups: Control, PRPH-MNs, PRPH-MNs+Rhein, PRPH-MNs+Rhein+NIR.


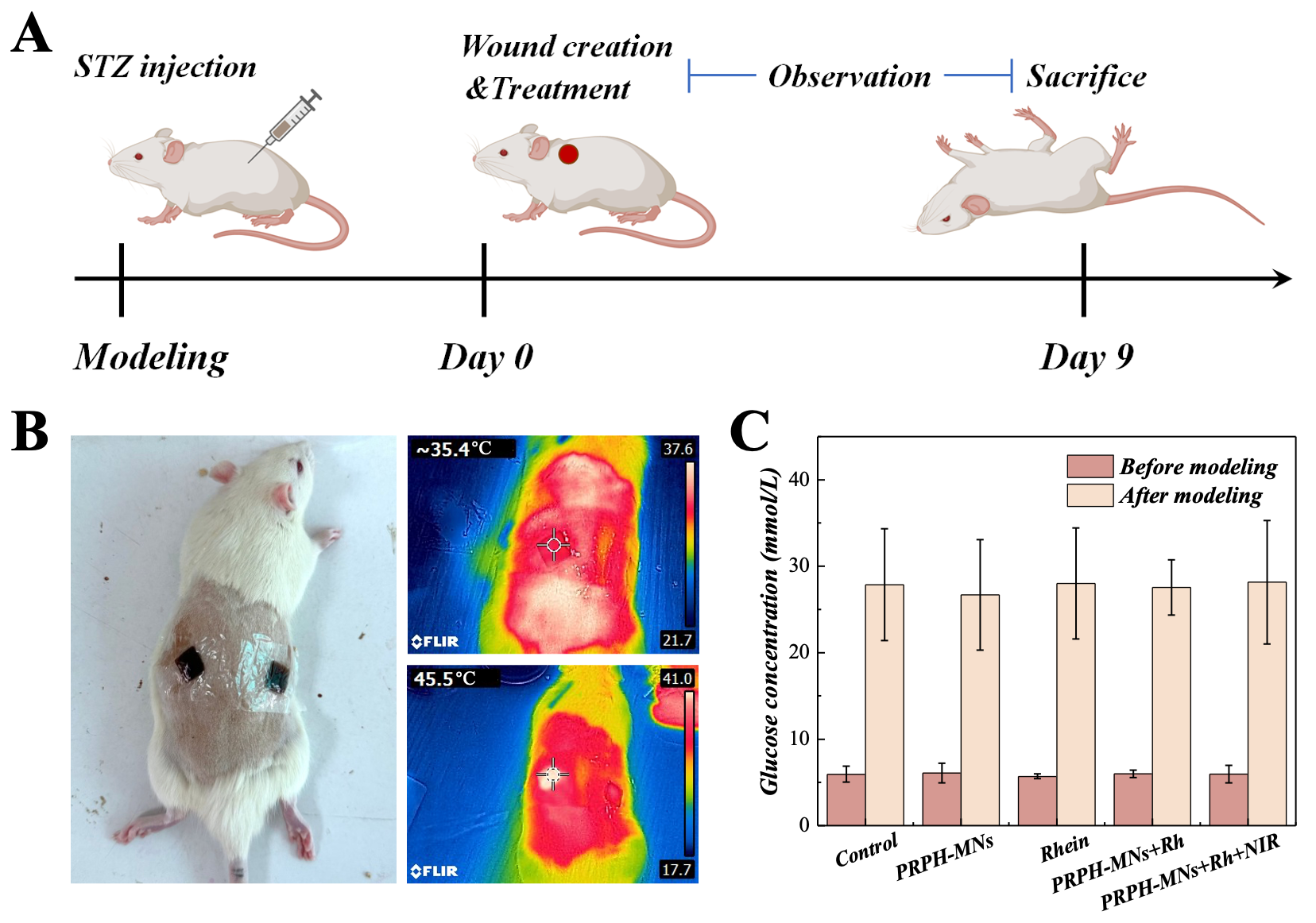


**Figure S8.** (a) Schematic illustration of experimental procedure for wound repair in diabetic rats. (b) PRPH-MNs+Rhein+NIR application in diabetes rat and infrared thermal images of its warming process. (c) Blood glucose concentration test of rats before and after diabetes modeling in five groups: Control, PRPH-MNs, Rhein, PRPH-MNs+Rhein, PRPH-MNs+Rhein+NIR.
